# Super-selective and staged glue embolization for labral AVMs using ultra-thin microcatheters: report of two cases

**DOI:** 10.1186/s42155-022-00335-w

**Published:** 2022-11-17

**Authors:** Miyuki Sawano, Shuichi Tanoue, Norimitsu Tanaka, Masamichi Koganemaru, Asako Kuhara, Tomoko Kugiyama, Yasumoto Shinjo, Toshi Abe

**Affiliations:** Department of Radiology, Kurume University School of Medicine, 67 Asahi-machi, Kurume, Fukuoka, 830-0011 Japan

**Keywords:** Arteriovenous malformation, Staged, Transcatheter arterial embolization, Super-selective, N-butyl-2-cyanoacrylate

## Abstract

**Background:**

Treating arteriovenous malformation (AVM) is challenging because of the high recurrence rate and because incomplete resection or embolization can induce aggressive growth. However, a standard strategy is not fully established. Although transcatheter arterial embolization (TAE) is currently almost always part of the treatment, in many cases, single treatment is not curative and only palliative. Additionally, the success and complication rates associated with TAE alone are unclear, and there has been limited study of staged TAE for facial AVMs. Furthermore, few reports have described the details of the procedure.

**Case-presentation:**

We report two cases of AVM of the upper lip in patients who were successfully treated by staged super-selective TAE at several-month intervals using ultra-thin microcatheters and n-butyl-2-cyanoacrylate.

**Conclusion:**

Staged and super-selective TAE may prevent complications and provide high curability and might be a useful treatment in cases of AVM.

## Introduction

**Fig. 1:**
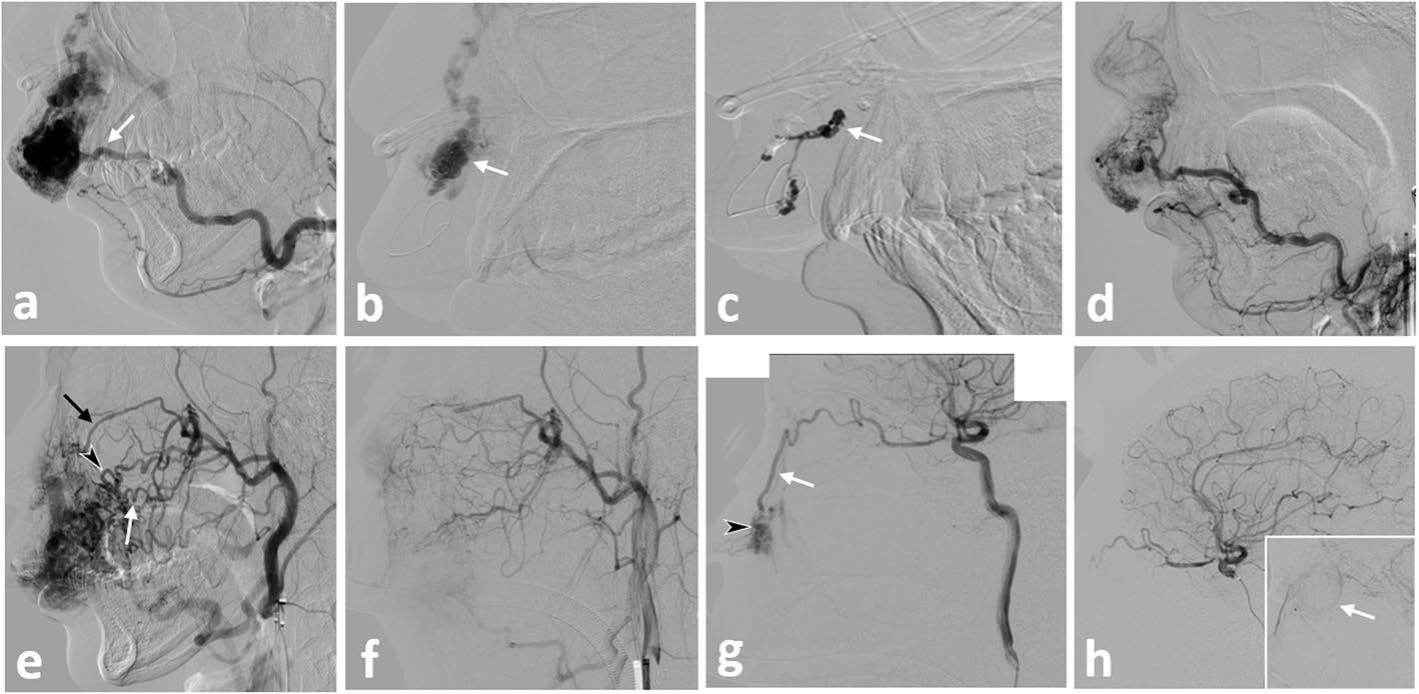
Angiograms in Case 1 showing high-flow AVMs with multiple shunts between the arterioles and venules as a complex vascular network (Cho’s classification type IIIb) in the left upper lip and cheek. The AVMs were fed by bilateral FAs and the left ICA and drained into bilateral facial and angular veins. **a** Right facial arteriogram, lateral view, before the first embolization session. Arrow indicates the right SLabA. **b** Pre-embolization left external carotid arteriogram before the first session. The microcatheter was navigated to the distal feeder from the right SLab A (arrow). **c** DSA, lateral view, during injection of nBCA under flow control. The nBCA-lipiodol mixture filled the shunting points and the distal draining veins (arrow). **d** Left external carotid arteriogram after embolization of the right FA after the second session showing devascularization of the AVM. **e** External carotid arteriogram, lateral view, after embolization of bilateral Slab As showing residual AVMs fed by the IOA (black arrow), greater palatine artery (white arrow), and transverse FA (arrowhead). **f** Left external carotid arteriogram after embolization of the left IOA from the left MA during the fifth session showing disappearance of the AVM. **g** Pre-embolization left internal carotid arteriogram during the fifth session. Arrow indicates the angular (lateral nasal) artery originating from the ophthalmic artery. Microcatheter was navigated into distal segment of the feeding artery (arrowhead). **h** Post-embolization after the fifth session. Left internal carotid arteriogram showing disappearance of the AVM without distal embolism into the cerebral artery and retinal artery (arrow: preserved retinal blush on a magnified image during the venous phase)

Arteriovenous malformation (AVM) is a rare vascular anomaly. Approximately half of extracranial AVMs occur in the head and neck (Fernández-Alvarez false, 2020). Various approaches, namely surgical resection, endovascular treatment, percutaneous embolization/sclerotherapy, and combination therapies have been used to manage AVMs (Melia false, 2017). However, a standard treatment strategy has not been established. The treatment of lip AVMs is associated with additional technical difficulties owing to the cosmetic and functional issues, risk of neurological and skin/mucosal complications, and the distant peripheral and fine angioarchitecture. A limited number of reports have described successful treatment using transarterial embolization (TAE) or percutaneous sclerotherapy for lip/facial AVMs (Melia false, 2017; Liu false, 2010; Juszkat false, 2009; Hayashi & Mochizuki, 2017; Griauzde false, 2020). This report presents two cases of AVM of the upper lip in patients successfully treated by staged TAE using a mixture of n-butyl-2-cyanoacrylate (n-BCA, B Braun, Melsungen, Germany) dissolved in Lipiodol (Guerbet, Paris, France) at several-months intervals. Using this approach, we were able to avoid post-procedure complications (Fig. 1).

## Case report

### Case 1

**Fig. 2:**
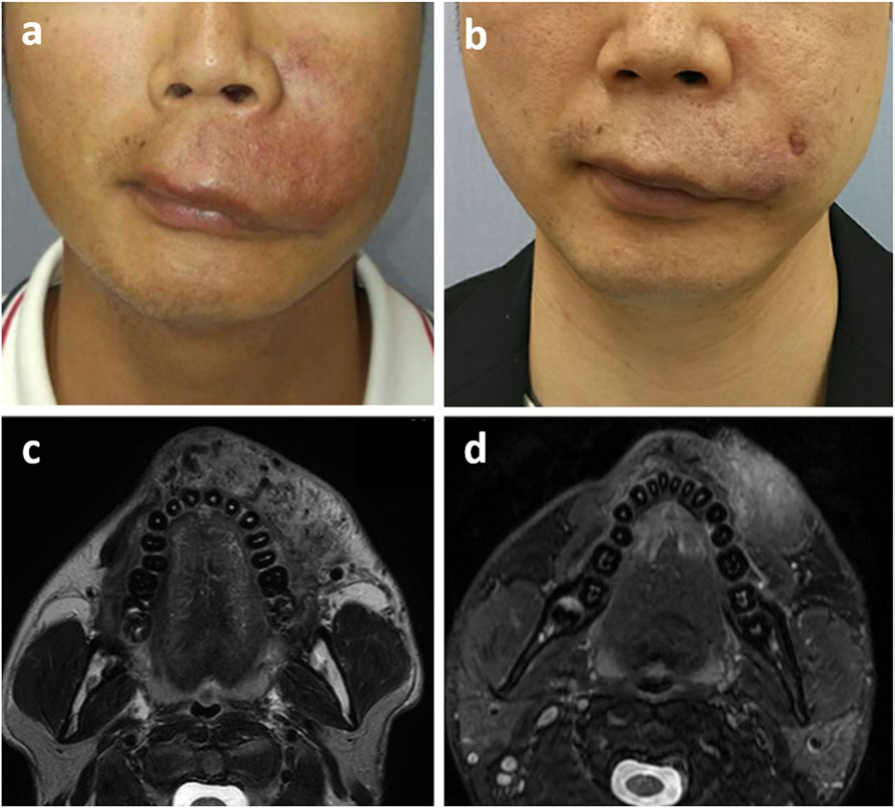
(**a**) Frontal view of the patient’s face in Case 1 before treatment showing swelling and erythema of the left upper lip and cheek, but no ulceration. (**b**) Although a small erosion occurred about a week after treatment then a scar remained on the left upper lip, swelling and erythema of the lip and cheek had resolved 6 months after the fifth embolization session. (**c**) T2WI MRI before treatment showing hyperintensity of the soft tissues, which were permeated by massive flow voids representing enlarged feeding and draining vessels. (**d**) Six months after the final session, fat-suppressed T2WI showing regression of the soft tissue swelling and disappearance of the flow voids

A 29-year-old man visited our department with a complaint of swelling of the upper lip and left cheek. He underwent partial resection after a diagnosis of hemangioma in his late teens. However, a few years later, the lesion gradually increased in size with swelling and erythema of the left upper lip and extension to the cheek, and he was referred to our hospital for treatment. Digital subtraction angiography revealed high-flow AVMs involving the left upper lip and cheek which belong to Schobinger’s stageII. The AVMs were fed by bilateral facial arteries (FA), and the left infraorbital artery and left ophthalmic artery, and the AVMs drained into bilateral facial and angular veins. The lesion was diagnosed in accordance with Cho’s classification as type IIIb AVM, indicating multiple shunts between the arterioles and venules, with dilated fistulae (Cho false, 2006). We performed five separate TAE sessions at approximately 4-month intervals with under conscious sedation using dexmedetomidine or general anesthesia. A 5-French guiding sheath (Fubuki: Asahi Intecc, Tokyo, Japan, or Axcelguide: medikit, Tokyo, Japan) was inserted via bilateral femoral arteries simultaneously, and the sheath was advanced into the bilateral external carotid artery (ECA). A 5-F balloon catheter (Cello; Medtronic, Irvine, CA, USA) was placed in the proximal ECA or other feeding arteries for flow control. Thin microcatheters with a 1.5-F tip (Marathon; Medtronic) or 1.3-F tip (DeFrictor; Medicos Hirata, Tokyo, Japan) and microwires (0.010″ TENROU: Kaneka Medics, Osaka, Japan or 0.008″ CHIKAI: Asahi Intecc, Tokyo, Japan) were coaxially navigated via the balloon catheter into the feeding artery as far distally as possible. A low-concentration n-BCA-lipiodol mixture was injected through the microcatheter with plug and push technique under flow control using balloon catheter until filling of the draining veins adjacent to the shunted points was achieved. The balloon catheters were positioned at the proximal segment of the bilateral ECA to prevent the retrograde collateral flow via the surrounding arteries and to obtain the sufficient penetration of the n-BCA into the draining venous segments. The embolized feeders in each session were as follows: left infraorbital artery, first session (25% n-BCA) and fifth session (20% n-BCA); left transverse FA and left FA, third session (20% n-BCA); left superior labial artery and lateral nasal artery from the left FA, fourth session (17% n-BCA); and left angular (lateral nasal) artery from the left ophthalmic artery, fifth session (20% n-BCA). After the final embolization session, left ECA angiography showed almost complete disappearance of the AVMs (Fig. 1). No complications, such as skin/mucosal necrosis or nerve injury or even any functional deficits of the lip occurred other than transient exacerbation of swelling, erythema and small erosion only required topical nonsteroidal anti-inflammatory drugs. Three months after the final session, T2-weighted magnetic resonance imaging showed regression of the previous soft tissue swelling and disappearance of the flow voids. Swelling and erythema of the lip and cheek had also resolved (Fig. 2). There was no regrowth during 20 months of follow-up after the last embolization treatment.

### Case 2

**Fig. 3:**
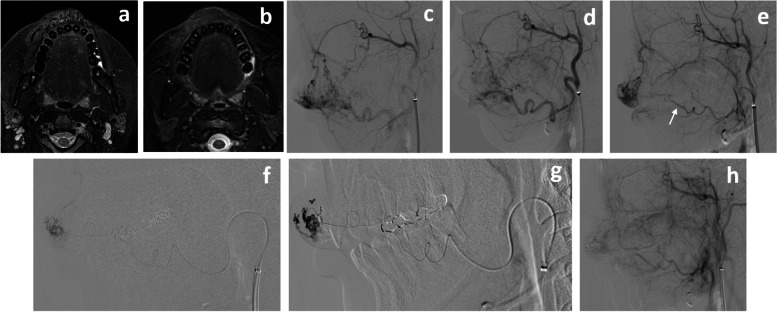
Angiogram in Case 2 showing high-flow AVMs in the right upper lip that were fed mainly by mainly bilateral SLab As from bilateral FAs and which drained into the right facial and angular veins. The lesion was diagnosed in accordance with Cho’s classification as type IIIa AVM. (**a**) Fat-saturated T2WI MRI showing high-signal intensity with multiple flow voids in the upper lip before treatment. (**b**) Fat-saturated T2WI MRI 3 months after the last embolization session showing regression of the soft tissue swelling and disappearance of the flow voids. (**c**) Right external carotid arteriogram, lateral view, before the first embolization session. (**d**) Right external carotid arteriogram showing partial devascularization of the AVM after embolization of the right SLab A during the first session. (**e**) Left external carotid arteriogram, lateral view, before the second embolization session showing residual AVMs fed by the SLab A (white arrow). (**f**) The microcatheter was navigated into the distal feeder from the left SLab A in the second embolization session. (**g**) Angiogram, lateral view, showing the nBCA injection under flow control. The nBCA-lipiodol mixture filled the shunting points and the distal draining veins. (**h**) Left external carotid arteriogram showing that the AVMs near-completely disappeared after embolization of the left SLab A during the second session

A 23-year-old female was referred to our department for further examination and treatment of Schobinger’s stageIIAVM of the upper lip. She had experienced swelling of the upper lip for at least 10 years. Diagnostic angiography revealed type IIIa AVM in the right upper lip that was fed mainly by bilateral superior labial arteries branching from bilateral FAs and which drained into the right facial and angular veins. Three embolization procedures were performed at 3–5-month intervals. All embolotherapy sessions were performed as follows under local anesthesia: A 5-French guiding sheath (Fubuki: Asahi Intecc, Tokyo, Japan or Axcelguide: Medikit, Tokyo, Japan) was inserted via bilateral femoral arteries simultaneously, and the sheath was advanced into bilateral ECAs. A 5-F balloon catheter (Medtronic) was placed in the proximal ECA for flow control. A thin microcatheter with a 1.3-F tip (DeFrictor; Medicos Hirata) and microwires (0.010″ TENROU; Kaneka Medics, Osaka, Japan or 0.008″ CHIKAI; Asahi Intecc, Tokyo, Japan) was coaxially navigated via the balloon catheter into the feeding artery as far distally as possible. A 20% nBCA-lipiodol mixture was injected through the microcatheter with plug and push technique under arterial balloon occlusion until filling of the draining veins close to the shunted points was achieved. The embolized feeders in each session were as follows: right (rt.) superior labial artery, first session (20% n-BCA); left superior labial artery, second and third session (20% n-BCA). Subsequent carotid arteriography showed devascularization of the AVM (Fig. 3). No complications or functional deficits occurred. Follow-up magnetic resonance 3 months after the last TAE session showed regression of the previous labral swelling and disappearance of the flow voids.

## Discussion

The treatment of AVM remains challenging owing to the high rate of recurrence, which is reported in up to 80% of patients (Fernández-Alvarez false, 2020). Moreover, incomplete resection and embolization can induce aggressive growth of the remaining lesion with the risk of progression in up to 50% of patients (Fernández-Alvarez false, 2020). Although a combination of embolization and surgical resection is the treatment of choice (Fernández-Alvarez false, 2020), a globally-approved treatment strategy has not been established.

Cho et al. (Cho false, 2006) developed an angiography-based classification system and proposed that type II AVMs were the most curable. In contrast, type IIIa and IIIb AVMs were more difficult to treat and were prone to higher failure and recurrence rates. The authors also reported high efficacy with ethanol embolization for AVMs, which resulted in cure or partial remission in 74% of cases; however, the complication rate ranged from 0% to 48% (Cho false, 2006). Another study reported complication rates with a combination of TAE and direct percutaneous puncture sclerotherapy for head and neck AVM ranging from 3% to 17% (Griauzde false, 2020). Ethanol has been reported to be the most efficient agent but cause serious complications especially of the superficial tissue. Although Onyx has a property of much less risk of immediate adhesion of the catheter and the longer solidification time, differing from n-BCA, is not commercially available for the treatment of peripheral AVMs in this country (indicated only for preoperative embolization of cerebral arteriovenous malformations and transvenous embolization of dural arteriovenous fistulas). Therefore, we selected n-BCA as embolic material for treatment.

The complications associated with AVM therapy comprise mucosal necrosis requiring surgical repair, permanent nerve injury, and transient subcutaneous edema (Griauzde false, 2020; Tiwari & Singh, 2015). Many studies have reported mucosal necrosis or nerve injury as minor complications, which can be managed conservatively (Juszkat false, 2009). However, standardized rates of functional impairment or other minor complications are unknown. Multi-stage procedures are often performed when treating AVMs to prevent excessive damage to local tissue and to contain recurrent disease early. However, multi-staged embolotherapy has not yet been reported to lower the rate of recurrence (Fernández-Alvarez false, 2020).

The main goal of AVM treatment is to occlude the arteriovenous shunts. Embolization of proximal arteries may lead to lesional growth with the development of collateral flow, which makes further interventions difficult. For successful embolization, it is essential to deliver embolic agents properly into both the shunting points and the draining veins, sparing the normal arterial branches supplying adjacent areas. The use of ultra-thin catheters allows approaching the distal feeding arteries, and injecting n-BCA from the level closest to the shunted points might result in high curability.

Few reports of the successful use of staged TAE for head and neck AVMs have been published, although a few case reports have described the details of the procedure (Melia false, 2017; Liu false, 2010; Juszkat false, 2009; Hayashi & Mochizuki, 2017; Griauzde false, 2020). Some case reports reported complete cures with no recurrences after surgical resection or after combined treatments comprising embolization and surgery (Tiwari & Singh, 2015; Martin false, 2006; Jafarian false, 2016). In these series, the lips required reconstructive repair after resection of the AVMs to address morphological and functional defects. In our cases, near-complete obliteration of the labial AVMs was achieved by staged super-selective glue embolization using ultra-thin microcatheters. The patients experienced no complications, such as ischemic necrosis of the skin and mucosa or functional deficits of the lip.

## Conclusion

Staged TAE might be safe thanks to the intervals between sessions, which allow for repair of any tissue damage and prevention of irremediable conditions. Staged TAE may prevent complications and provide high curability and might be a useful treatment in cases of AVM.

## Abbreviations

AVM: Arteriovenous malformation
TAE: Transcatheter arterial embolization
nBCA: n-butyl-2-cyanoacrylate
FA: Facial artery
ECA: External carotid artery
ICA: Internal carotid artery
rt. Slab: A right superior labial artery
DSA: Digital subtraction angiography
IOA: Infraorbital artery
MA: Maxillary artery
T2WI MRI: T2-weighted magnetic resonance imaging
